# A novel ligand on astrocytes interacts with natural cytotoxicity receptor NKp44 regulating immune response mediated by NK cells

**DOI:** 10.1371/journal.pone.0193008

**Published:** 2018-02-15

**Authors:** Kelly E. Bowen, Stephen O. Mathew, Kathleen Borgmann, Anuja Ghorpade, Porunelloor A. Mathew

**Affiliations:** Department of Microbiology, Immunology and Genetics, University of North Texas Health Science Center, Fort Worth, Texas, United States of America; Emory University School of Medicine, UNITED STATES

## Abstract

NK cells play important role in immunity against pathogens and cancer. NK cell functions are regulated by inhibitory and activating receptors binding corresponding ligands on the surface of target cells. NK cells were shown to be recruited to the CNS following several pathological conditions. NK cells could impact CNS physiology by killing glial cells and by secreting IFN-γ. Astrocytes are intimately involved in immunological and inflammatory events occurring in the CNS and reactive astrogliosis is a key feature in HIV-associated neurocognitive disorders. There is little data on NK-astrocyte interactions and ligands expressed on astrocytes that could impact NK cell function. Natural cytotoxicity receptors (NCRs) play a critical role in the cytolytic function of NK cells. Among the NCRs, NKp44 is unique in expression and signal transduction. NKp44 is expressed only upon activation of NK cells and it can mediate both activating and inhibitory signals to NK cells. Here, we have studied the expression and function of natural cytotoxicity receptor NKp44 upon NK-astrocytes interactions in the presence or absence of an HIV peptide (HIV-3S peptide) shown to induce NK cell killing of CD4+ T cells during HIV–infection. Using a fusion protein consisting of the extracellular domain of NKp44 fused to Fc portion of human IgG, we determined the expression of a novel ligand for NKp44 (NKp44L) on astrocytes. Incubation of astrocytes with HIV-3S peptide downregulated NKp44L expression on astrocytes implicating protection from NK mediated killing. Thus, our study showed that NKp44 have a protective effect on astrocytes from NK cell mediated killing during HIV infection and impact astrocyte role in HAND.

## Introduction

The human immunodeficiency virus (HIV-1) can invade the central nervous system (CNS) after primary infection and infect CNS resident cells, such as astrocytes. HIV-1 infected CNS cells results in inflammatory responses generated in the CNS, leading to long-term neuroinflammation and neuronal damage [1]. This neuronal damage can cause neuropsychological deficits, collectively referred to as HIV-associated neurological disorders (HAND) [2]. Since, both HIV-1 binding and infection can affect astrocyte function, astrocytes have a strong pathogenic potential for being intimately involved in HAND [3]. HIV-1 infection of astrocytes also damages the blood brain barrier (BBB) which can lead to recruitment of natural killer (NK) cells to the CNS [4].

NK cells are granular lymphocytes that play a vital role in defense against viral infections and cancer. NK cells survey host tissues and kill abnormal cells or virally infected cells [5, 6]. The majority of NK cells are localized in peripheral blood, lymph nodes, spleen and bone marrow but can be induced to migrate toward inflammation site by different chemoattractants [7]. NK cell function is regulated by a balance between activating and inhibitory signals transmitted through NK cell surface receptors upon interaction with their ligands. Their functions include: release of cytotoxic granules, antibody-dependent cell-mediated cytotoxicity (ADCC), and cytokine production [8, 9]. NK cells work to control viral infections by secreting IFN-γ and TNF-α [5, 10, 11]. NK cells undoubtedly play a role in the immune response against HIV-1. NK cells can limit HIV replication through direct killing of infected cells as well as the secretion of anti-viral cytokines and chemokines that suppress HIV-1 replication [12, 13]. NK cells from HIV patients show a functional impairment to kill tumor cells, a possible explanation for the increase in opportunistic tumors in HIV patients [13]. Studies have also shown that HIV-1 exposed but not infected individuals showed an increase in NK cell function suggesting a protective effect [14, 15]. Conversely, HIV decreases the expression of natural cytotoxicity receptors (NCRs), overall decreasing NK cell activation [13, 16]. Expression of NK activating receptor KIR3DS1 in combination with HLA-B allele is associated with delayed progression to AIDS and KIR3DS1 in the absence of HLA-B allele is associated with more rapid progression to AIDS [17]. Not only is NK cell receptor expression altered during HIV-1, their ligand expression can also be altered. HIV induces the NKG2D ligands and downregulates CD48 ligand [18]. The cell-cell interactions of NK cells and HIV-1 infected astrocytes especially in the context of HAND are understudied.

Natural cytotoxicity receptor NKp44 (CD336) is only expressed on activated NK cells. IL-2 induces the expression of NKp44 on NK cells [19]. NKp44 can be activating or inhibitory depending on the ligand it binds [20, 21]. Strikingly, NKp44L has not yet been detected on circulating cells isolated from healthy individuals, but it is expressed on a large panel of the tumor and transformed cells [22, 23]. The known cellular activating ligand of NKp44 (NKp44L) is an isoform of the mixed-lineage leukemia-5 protein (MLL5) [22, 23]. Its activating ligand is expressed in numerous tumor and transformed cell lines rendering them more sensitive for NK cytotoxicity. Previous studies in our lab identified, PCNA/HLA-1 as an inhibitory ligand for NKp44 [24].

NKp44 and its ligand, NKp44L, have strong implications in HIV-1 infection. A substantial percentage of NK cells from HIV-1 patients express the NKp44. Stimulation with HIV-3S peptide has been proven to induce the expression of NKp44L in both infected and uninfected CD4 T cells, resulting in their lysis by NK cells [14]. Following traumatic, infectious, and autoimmune-mediated brain injury, NK cells have been found in the CNS, but the functional significance of NK cell recruitment and their mechanisms of action during brain inflammation are not well understood. NK cell function in the CNS following brain injury could either be neuroprotective or neurotoxic [4]. A very few studies have investigated NK cell interactions with astrocytes. The few studies that have been conducted, have shown NK cells are capable of killing astrocytes and that IL-2 augments NK cell killing of astrocytes [19].

Our study aimed to identify important molecules involved in NK cell signaling on astrocytes that allow for NK directed cytotoxicity of astrocytes and compare NK cell killing of astrocytes in the presence and absence of HIV-3S peptide (Ac-SWSNKS-NH2). Using a fusion protein consisting of the extracellular domain of NKp44 fused to Fc portion of human IgG, we determined the expression of a ligand for NKp44 on astrocytes. Incubation of astrocytes with HIV-3S peptide downregulated NKp44L expression on astrocytes implicating protection from NK mediated killing. Thus, our study showed that NKp44 has a protective effect on astrocytes from NK cell mediated killing during HIV infection, suggesting a viral escape mechanism.

## Materials and methods

### Isolation and culturing of primary human fetal astrocytes

Primary human fetal astrocytes were obtained from Dr. Ghorpade’s lab, University of North Texas Health Science Center, Fort Worth, TX. Human astrocytes were isolated from first and early second trimester elected aborted specimens as previously described [25]. Briefly, tissues ranging from 82 to 127 days were procured in full compliance with the ethical guidelines of the National Institutes of Health, University of Washington and North Texas Health Science Center. Cell suspensions were centrifuged, washed, suspended in media, and plated at a density of 20 × 10^6^ cells/150 cm^2^. The adherent astrocytes were treated with trypsin and cultured under similar conditions to enhance the purity of replicating astroglial cells. These astrocyte preparations were routinely >99% pure as measured by immunocytochemistry staining for glial fibrillary acidic protein (GFAP).

### Cell culture

NK92-MI cells (ATCC CRL-2408), were grown and cultivated in minimum essential medium (MEM) alpha medium (Life Technologies, Carlsbad, CA) supplemented with 12.5% FBS and donor horse serum (Atlanta Biologicals, Lawrenceville, GA), 0.2 mM inositol, 0.1 mM 2-mercaptoethanol, and 0.02 mM folic acid. All cells were cultured at 37°C in a humidified 5% CO_2_/95% air environment. Peripheral blood mononuclear cells (PBMCs) were isolated from ethylene-diamine-tetra-acetic acid (EDTA)-treated whole-blood samples by Histopaque-1077 (Sigma Chemicals, St. Louis, MO) density gradient centrifugation using LeucoSep tubes (Greiner, Monroe, NC) from healthy individuals with prior approval from the Office of Protection of Human Subjects, The North Texas Regional Institutional Review Board (IRB# 20–28) of UNT Health Science Center, Fort Worth, TX. A written consent was obtained from healthy donors and no minors were involved in this study. Primary NK cells were isolated from the PBMCs using NK isolation kit (Miltenyi Biotec, San Diego, CA) and the purity was determined by flow cytometry using anti-human CD56 mAb.

### Construction and expression of Soluble NKp44-Ig fusion protein

**Fig 1:**
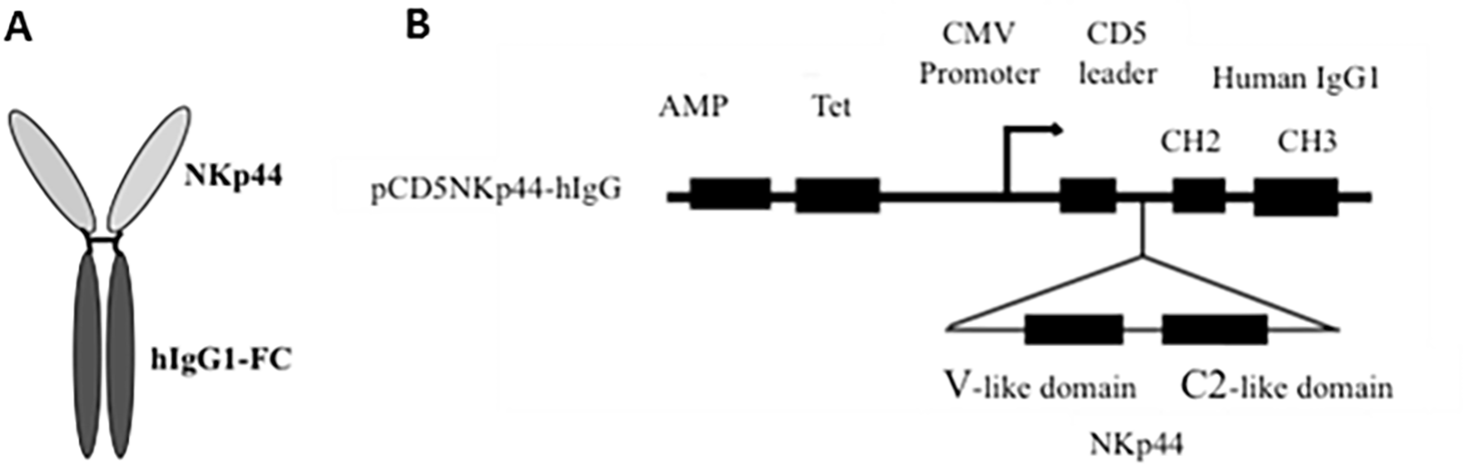
Schematic representation of the mammalian expression vector construct and the NKp44-Fc fusion protein. (A) Fusion protein is bivalent, containing two extracellular domains in the arm regions and one Fc region. (B) The extracellular domain of NKp44 was PCR amplified and subcloned in front of the CH2, CH3 domain of human IgG1 in frame with the CD5 leader sequence and CH2 as described in materials and methods.

Soluble NKp44-Ig fusion protein was produced by fusing the extracellular domain of NKp44 with the Fc portion of human IgG. The extracellular domain of NKp44 was amplified by PCR (forward primer NKp44*Nhe*I FP-59 TCGCTAGCGCAATCCAAGGCTCAGGT-39 and reverse primer NKp44*Bam*HIRP-59 CTCGGGATCCGTGTCTGCAGG GCCA-39). The amplified product was subcloned in front of human Fc gene at *Nhe*I and *Bam*H I cloning sites in pCD5 vector, which contained the CH2 and CH3 regions of the human IgG1. Soluble NKp44-Ig fusion protein was produced by transiently transfecting the plasmid into HEK-293 cells using Fugene-6 transfection reagent (Roche Diagnostic Corporation, Indianapolis, IN). Cells were cultured in Optimem I (Life Sciences, Carlsbad, CA) reduced serum free media during transfection and collection of supernatants. Supernatants collected on days 2 and 3 after transfection were centrifuged to remove cellular debris, and then concentrated to 1 mg/μl with a 35,000 molecular weight centrifugal concentrator. Concentrated supernatants were verified for the presence of functional fusion protein by flow cytometry and western blotting. Supernatants of untransfected HEK-293 cells cultivated in Optimem I media were concentrated and used as negative controls in flow cytometry utilizing NKp44 fusion protein. A dose response curve was generated to determine optimum binding of NKp44-Ig prior to fusion protein use, which determined 70 mg of NKp44-Ig at a concentration of 1 mg/μl produces the largest shift of peak fluorescence without oversaturation. Schematic representation of the NKp44-Ig fusion protein is shown in Fig 1.

### HIV-3S peptide stimulation

In order to determine whether HIV-3S peptide modulated the expression of receptors and ligands, cells were resuspended at 1 × 10^5^ cells/500 μl in Opti-MEM® I reduced serum medium (Life Technologies, Carlsbad, CA) plated in a 96 well round bottom plate with increasing concentrations of the HIV peptide Ac-SWSNKS-Nh2 (3S) (Celtek Peptides, Nashville, TN) at 1 μg/ml and 10 μg/ml added to different wells. The incubation period was 4 hours and took place at 37°C in a humidified 5% CO2/95% air environment. After incubation astrocytes were washed with PBS and detached with 0.25% trypsin-EDTA.

### Flow cytometry

Astrocytes treated with 10 μl/ml HIV-3S peptide or astrocytes that received no HIV-1 3S peptide stimulation were resuspended in 500 μl PBS-BSA buffer (PBS, 1% bovine serum albumin) and first incubated with Human IgG Fc fragment (Rockland, Gilbertsville, PA) to block nonspecific interactions with Fc receptors. To test whether HIV-3S peptide induced the expression of NKp44L, 70 μg of NKp44-Ig fusion protein was added to 1 × 10^6^ FC blocked astrocytes and Jurkat lymphocyte cells that received 1μg/ml HIV-1 3S peptide, 10 μl/ml HIV-1 3S peptide, and cells that received no peptide stimulation [24]. Astrocytes and Jurkat cells were incubated with the fusion protein on ice for 45 minutes prior to incubation with the secondary antibody. After fusion protein incubation, the cells were washed twice with buffer and then incubated with 2.5 μg of the secondary antibody, anti-hIgG-Fc-PE (Biolegend, San Diego, CA) for 30 minutes and analyzed by a Beckman Coulter Cytomics FC500 flow cytometer. Cells incubated with the secondary antibody alone with no NKp44-Ig fusion protein were used as negative controls.

### ^51^Cr release assay

The killing of astrocytes by blocking NKp44 interactions or CD38 interactions on NK cells was tested using a Chromium-51 radionuclide (^51^Cr) release assay. Killing assays were done on astrocytes with 10 μg/ml HIV-3S and astrocytes with no peptide stimulation using NK92-MI cells or primary NK cells as the effector cells. Astrocytes and NK cells were incubated with human IgG Fc fragment to block interactions with Fc receptors on both cells prior to use. Cells were resuspended in their corresponding media. Astrocytes were incubated with ^51^Cr (PerkinElmer, Waltham, MA) for 90 mins at 37°C in a humidified 5% CO_2_/95% air environment. ^51^Cr labeled astrocytes were then treated with 15 μg of NKp44 fusion protein, or 1 μg mIgG1 isotype. NK92-MI cells were previously confirmed to express NKp44 after two weeks of culture. To block NKp44 and CD38 on NK cells, NK cells were incubated with 2.5 μg anti-CD38 or anti-NKp44 or mIgG1 isotype. Astrocytes were then incubated with NK92-MI at effector to target ratios of 20:1, 10:1, and 5:1 for 4 hours at 37°C. Percent specific lysis was compared to astrocytes incubated with 0.5 mg/ml mIgG1 isotype antibody or no antibody, which served as a positive control (No Blocking) of cell lysis under unblocked conditions. Alternatively, NK92-MI cells were incubated with 0.5 mg/ml anti-NKp44 or mIgG1 isotype control antibody prior to incubation with astrocytes incubated with no antibody. Supernatants were collected and percent specific lysis was calculated. Experiments were performed in triplicates.

### IFN-γ ELISA assay

NK92-MI cells and astrocytes were blocked with Fc fragment. NK92-MI cells were then treated with 2.5 μg anti-NKp44 or anti-CD38. NK92-MI cells with no antibody or with 2.5 μg mIgG1 were used as controls. Astrocytes with and without HIV-3S peptide stimulation were treated with 1 μg mIgG1 isotype control or received no antibody treatment. NK92-MI cells and astrocytes were incubated overnight in a 96 well plate such that the ratio of NK cells to astrocytes was 10:1. The supernatants were collected and plated for ELISA. 96 well ELISA plates were coated with 100 μl/well capture antibody and incubated overnight at 4° C. After several washings, biotin-conjugated detection antibodies plus HRP-conjugated streptavidin were added. After extensive washing, the substrate solution was added to each well and incubated for 30 min at RT in the dark. After adding 50 μl of stop solution, the optical density at 450 nm was determined; 570 nm was used as a correction wavelength.

## Results

### NKp44L is expressed on astrocytes and HIV-3S peptide downregulates its expression

**Fig 2:**
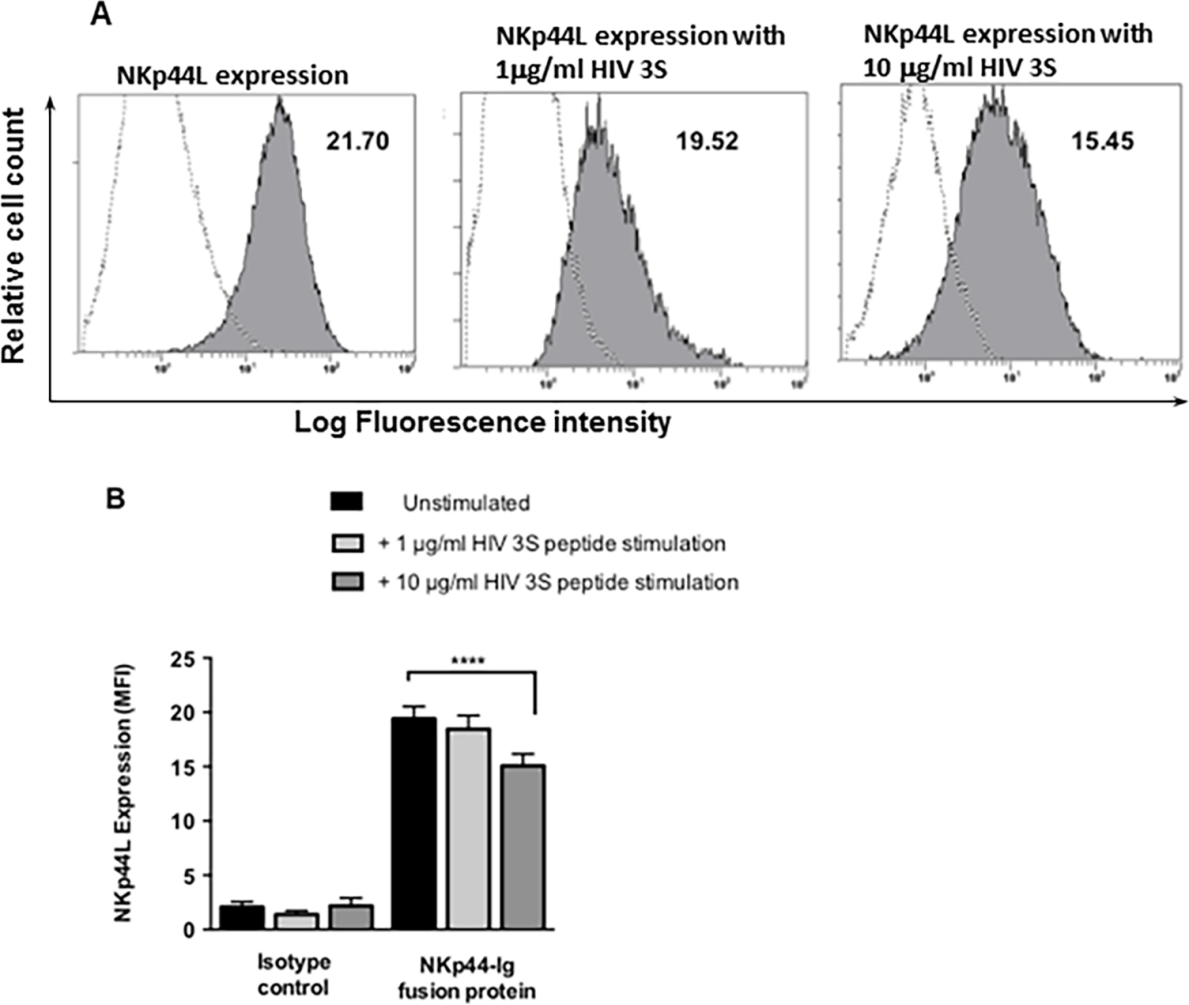
Astrocytes express a ligand for NKp44 and increasing concentrations of HIV 3S peptide decreases its expression. (A) The expression of a ligand for NKp44 (NKp44L) on astrocytes was established using 70 μg of NKp44-Ig fusion protein detected by anti-IgG-Fc-PE (filled histogram). Astrocytes incubated with 2.5 μg anti-IgG-Fc-PE were used as negative controls (open histogram). Cells were tested for the binding of NKp44-Ig fusion protein with no stimulation, 1 μg/ml peptide stimulation, and 10 μg/ml stimulation. Mean Fluorescent Intensity (MFI) values, the differences between the mean value for the control group and the experimental groups were quantified and MFI is indicated in the top right corner of all plots. Fig A is representative of 1 sample. (B) The combined MFIs of tests done in duplicate on 5 different astrocyte donors. Data are represented as means ± SEM, **** p < 0.0001, ANOVA.

We studied the expression and function of NCR NKp44 upon NK-astrocytes interactions in the presence or absence of an HIV-3S peptide using a fusion protein consisting of the extracellular domain of NKp44 fused to Fc portion of human IgG. HIV-3S peptide was shown induce NKp44L on T cells and increasing concentrations of HIV-3S peptide increased NKp44L expression [14]. We did similar studies using astrocytes and determined that astrocytes significantly express NKp44L. Incubation of astrocytes with increasing concentrations of HIV-3S peptide significantly downregulated NKp44L expression on astrocytes (Fig 2).

### Blocking of NKp44 interactions protects astrocytes from NK92-MI and primary NK cell killing

**Fig 3:**
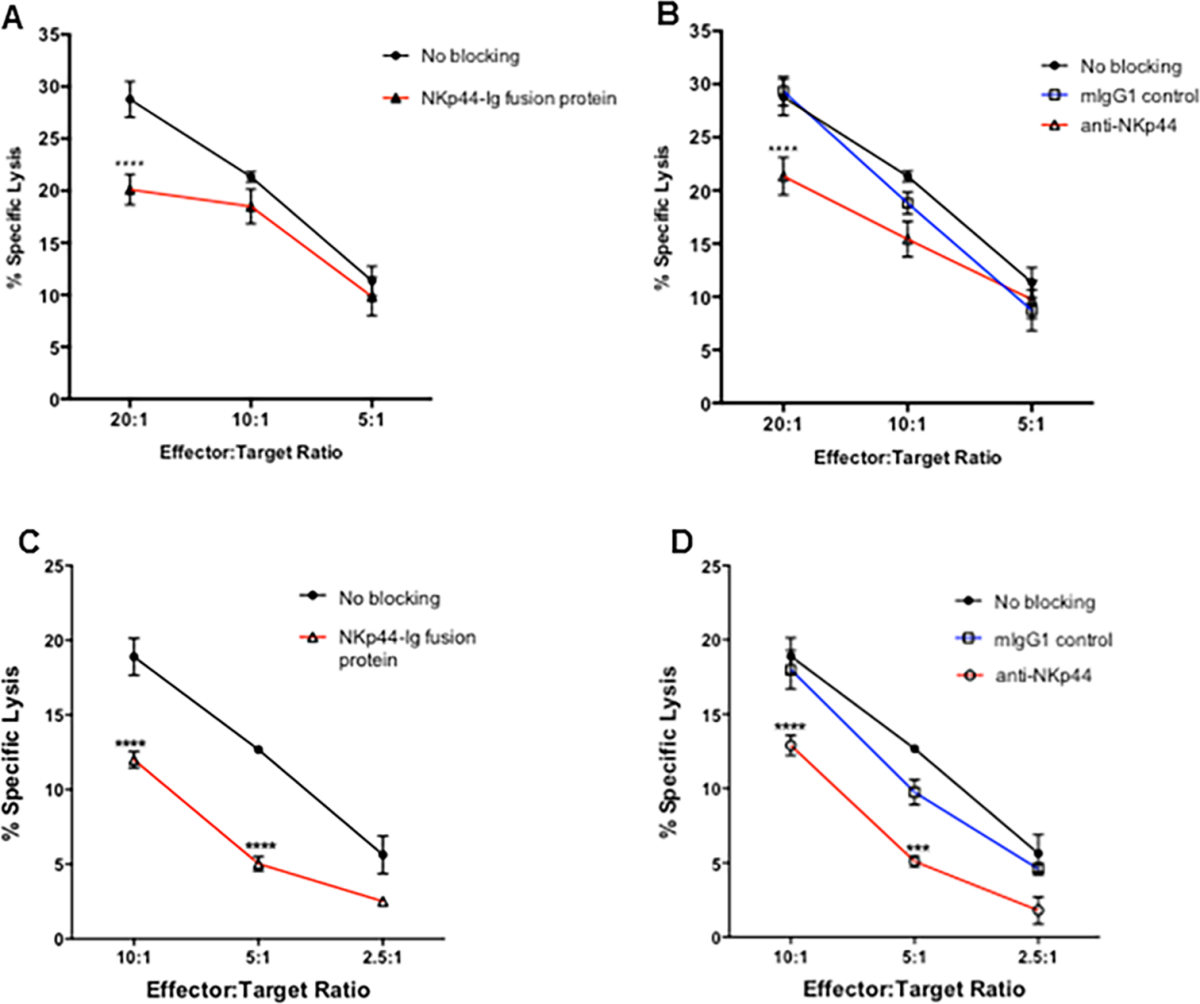
Blocking NKp44 interaction inhibits NK92-MI and primary NK cell mediated killing of astrocytes. NK92-MI and primary NK cell mediated lysis of astrocytes was determined using a standard ^51^Cr release assay. Astrocytes and NK92-MI cells (A & B) / primary NK cells (C & D) were first blocked with Human IgG Fc fragment to prevent reverse binding of fusion protein and antibody dependent cellular cytotoxicity. Astrocytes cells were labeled with ^51^Cr and incubated with 1 μg/μl of NKp44-Ig fusion protein (A, C). Astrocytes were then incubated with NK92-MI and primary NK cells at varying effector to target cell ratios for 4 hours at 37°C. Percent specific lysis of astrocytes was compared to astrocytes incubated with 0.5 mg/ml mIgG1 isotype antibody or no antibody, which served as a positive control (No Blocking) of cell lysis under unblocked conditions. Alternatively, NK92-MI and primary NK cells were incubated with 0.5 mg/ml anti-NKp44 or mIgG1 isotype control antibody prior to incubation with astrocytes incubated with no antibody (B, D). Figure is representative of 3 independent experiments performed in triplicate. Data is displayed as means ± SEM. ** *p* < 0.01, *** *p* < 0.001, **** *p* < 0.0001, ANOVA.

Our studies showed expression of a ligand for NKp44 on primary human fetal astrocytes. NKp44 interactions can be activating or inhibitory depending on the ligand it binds to [20, 21]. To establish if the ligand for NKp44 on astrocytes is inhibitory or activating, NK killing of astrocytes was tested with the blocking of NKp44 interactions and compared to the killing of astrocytes that did not receive any blocking antibodies. NKp44 interactions were blocked by either blocking NKp44 on NK cells using anti-NKp44 or by blocking NKp44L on astrocytes using NKp44-Ig fusion protein. Astrocytes were incubated with ^51^Cr and incubated with 1.0 μg/μl NKp44-Ig fusion protein (Fig 3A). Astrocytes were then incubated with NK92-MI cells at varying target to effector cell ratio. The percent specific lysis of astrocytes was compared to those cells that were incubated with no antibody, which served as a positive control (no blocking) of cell lysis under unblocked conditions. Alternatively, NK92-MI cells were incubated with 0.5 mg/ml anti-NKp44 or mIgG1 isotype control antibody prior to incubation with astrocytes incubated with no antibody (Fig 3B). Blocking of NKp44L on astrocytes decreased NK cell killing significantly at the 20:1 effector to target cell ratio when compared to cells without any blocking (Fig 3A). The blocking on NKp44 on NK cells also significantly decreased NK cell killing at the 20:1 effector to target cell ratio compared to cells that were treated with mIgG1 isotype control or cells that received no blocking treatment.

In order to determine whether blocking NKp44 interactions on primary human NK cells inhibits NK cell cytotoxic function, we isolated primary NK cells from PBMCs of healthy individuals. The primary NK cells were cultured in recombinant human IL-2. The experiments were conducted under the same specifications as for NK92-MI cells except the target to effector cell ratios were 10:1, 5:1, and 2.5:1. The blocking of NKp44L on astrocytes significantly decreased their lysis by on primary NK cells (Fig 3C) at the 10:1 and 5:1 effector to target cell ratio. The blocking of NKp44 on primary NK cells also significantly inhibited NK cell cytotoxic function when compared cells that received the isotype control or no blocking treatment (Fig 3D).

### HIV-3S peptide stimulation protects astrocytes and the blocking of NKp44 further protects HIV-3S stimulated astrocytes from NK92-MI and primary NK cell killing

**Fig 4:**
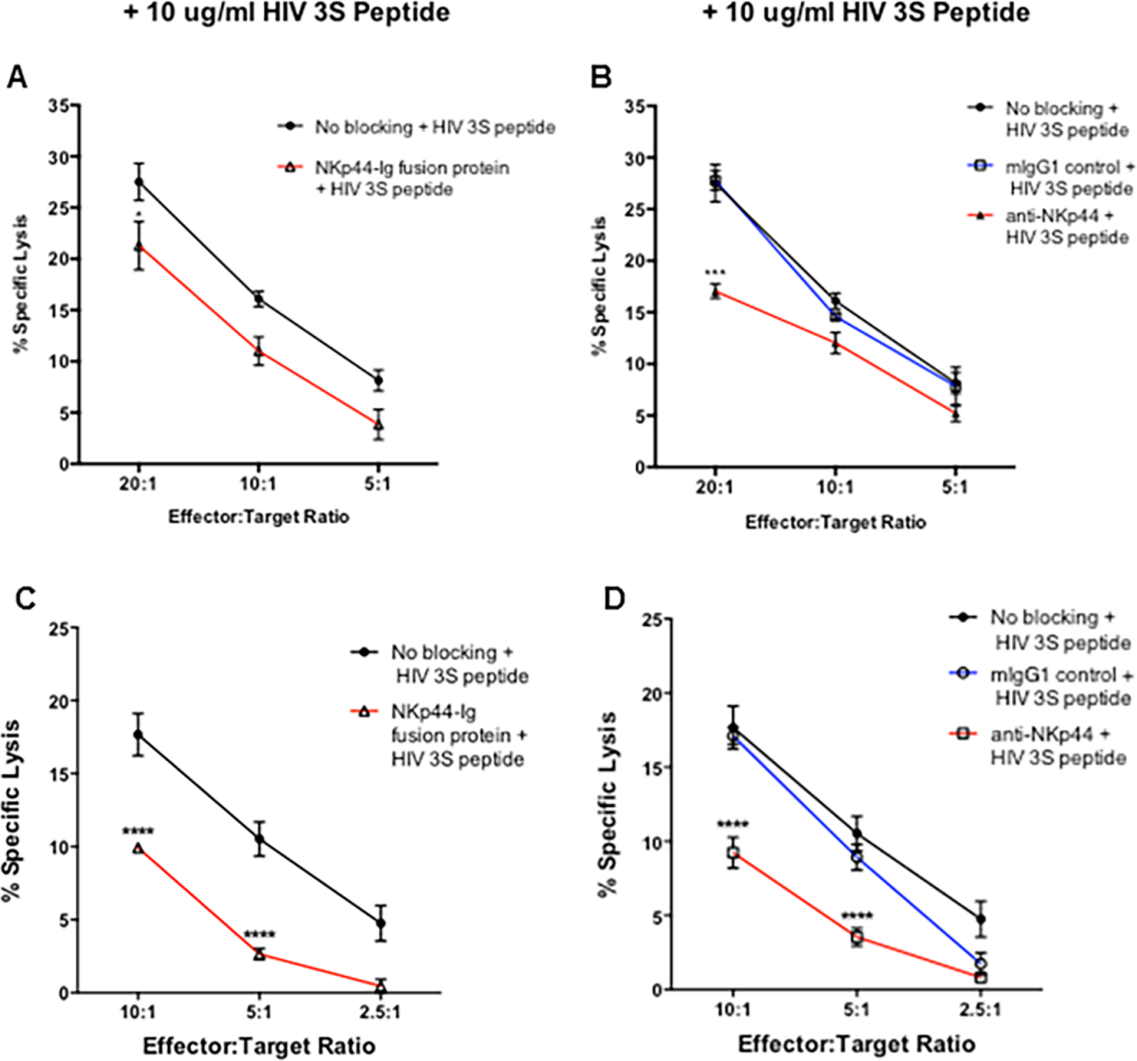
HIV-3S peptide stimulation protects astrocytes and the blocking of NKp44 further protects HIV-3S stimulated astrocytes from NK92-MI and primary NK cell mediated killing. Astrocytes were stimulated with 10 μg/ml HIV 3S peptide for 4 hours prior being labeled with ^51^Cr. NK92-MI and primary NK cell mediated lysis of astrocytes was determined using a standard ^51^Cr release assay. Astrocytes and NK92-MI cells (A & B) / primary NK cells (C & D) were first blocked with Human IgG Fc fragment to prevent reverse binding of fusion protein and antibody dependent cellular cytotoxicity. Astrocytes cells were labeled with ^51^Cr and incubated with 1 μg/μl of NKp44-Ig fusion protein (A, C). Astrocytes were then incubated with NK92-MI and primary NK cells at varying effector to target cell ratios for 4 hours at 37°C. Percent specific lysis of astrocytes was compared to astrocytes incubated with 0.5 mg/ml mIgG1 isotype antibody or no antibody, which served as a positive control (No Blocking) of cell lysis under unblocked conditions. Alternatively, NK92-MI and primary NK cells were incubated with 0.5 mg/ml anti-NKp44 or mIgG1 isotype control antibody prior to incubation with astrocytes incubated with no antibody (B, D). Figure is representative of 3 independent experiments performed in triplicate. Data is displayed as means ± SEM. ** *p* < 0.01, *** *p* < 0.001, **** *p* < 0.0001, ANOVA.

In order to observe whether blocking NKp44 interactions further protected HIV-3S peptide astrocytes from NK cell attack, astrocytes were stimulated overnight with 10 μg/ml HIV-3S peptide before being incubated with ^51^chromium. NKp44 interactions were blocked by blocking NKp44L on astrocytes (Fig 4A) or by blocking NKp44 receptor on NK92-MI cells (Fig 4B). Blocking NKp44 signaling significantly inhibited the lysis of HIV-3S peptide stimulated astrocytes (Fig 4A and 4B). Stimulation with HIV-3S peptide further protected astrocytes from NK92-MI cell killing as compared to unstimulated astrocytes.

Similarly to observe whether HIV-3S peptide decreased this inhibition on primary NK cells, astrocytes were stimulated overnight with 10 μg/ml HIV-3S peptide before being loaded with chromium and incubated with primary NK cells. Blocking of NKp44L on astrocytes (Fig 4C) or the blocking of NKp44 on primary NK cells (Fig 4D) also significantly inhibited NK cell lysis of astrocytes. HIV-3S peptide overall decreased NK cell lysis of astrocytes.

### Blocking NKp44 decreased IFN-γ production of NK- astrocyte co-cultures and the presence of HIV-3S peptide did not increase or decrease IFN-γ production of NKp44 blocked cells

**Fig 5:**
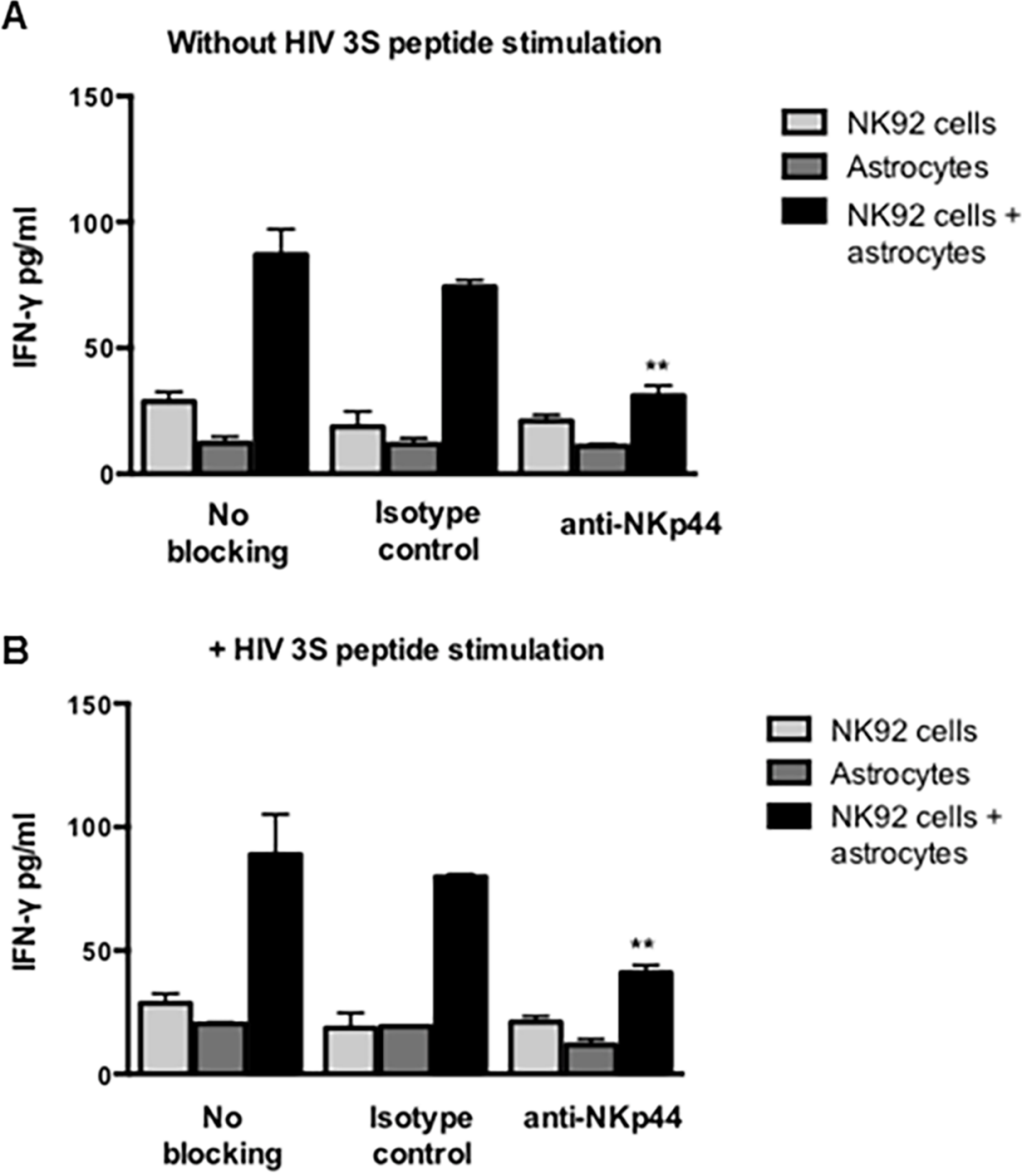
Blocking NKp44 interaction decreases the release of IFN-γ. IFN-γ levels were determined from astrocyte-NK cell co-culture supernatants by an IFN-γ ELISA. NK92-MI cells and astrocytes were first Fc blocked. NK92-MI cells were then treated with 2.5 μg anti-NKp44 and then incubated with astrocytes in the absence (A) and presence of HIV-3S peptide (B). NK92-MI cells with no antibody treatment or with 2.5 μg mIgG1 were used as controls. Astrocytes with and without HIV-3S peptide stimulation was treated with 1 μg anti-NKp44, mIgG1 isotype control or received no antibody treatment. Figure is representative of 2 independent experiments performed in triplicate. Bars ± SEM, ** *p* < 0.01, ANOVA.

IFN-γ production were determined from astrocyte-NK cell co-culture supernatants by an IFN-γ ELISA assay. NK92-MI cells and astrocytes were first Fc blocked. NK92-MI cells were then treated with 2.5 μg anti-NKp44 and then incubated with astrocytes at a ratio of 10:1 in the absence (Fig 5A) and presence of HIV-3S peptide (Fig 5B). NK92-MI cells with no antibody treatment or with 2.5 μg mIgG1 were used as controls. Astrocytes and NK92-MI cells treated with mIgG1 isotype control or received no antibody treatment were cultured alone and compared to the co-culture levels. Both NK cells can release IFN-γ. IFN-γ release of astrocytes and NK cells cultured alone with mIgG1 isotype control or no antibody treatment was quantified and these values were subtracted from the IFN-γ release of astrocytes and NK cells co-cultured together. Overall, NK92-MI cells incubating with astrocytes increased IFN-γ production. Blocking NKp44 on NK92-MI-astrocyte co-cultures decreased IFN-γ production (Fig 5A). Stimulation with HIV-3S peptide appeared to have no effect on IFN-γ even with NKp44 blocked (Fig 5B).

## Discussion

NK cells were shown to be recruited to the CNS following several pathological conditions such as HAND [4]. Because both HIV-1 binding and infection can affect astrocyte function, astrocytes have a strong pathogenic potential for being intimately involved in HAND. The interactions between NK cells and CNS glial cells, especially astrocytes, are understudied. NK cells could impact CNS physiology by killing glial cells and by secreting IFN-γ. Previous studies reported the ability of NK cells to kill astrocytes [19, 26, 27] but their mechanism of killing action was not previously investigated and no prior studies have investigated NK-astrocyte interactions in the context of HAND.

NCRs play a critical role in the cytolytic function of NK cells. Among the NCRs, NKp44 is unique in its expression and signal transduction. NKp44 is expressed only upon activation of NK cells and it can mediate both activating and inhibitory signals to NK cells [19]. The ligand for NKp44L has only been previously reported on infected or transformed cells [22, 23]. Using a fusion protein consisting of the extracellular domain of NKp44 fused to Fc portion of human IgG, we determined healthy human fetal astrocytes express a novel ligand for NKp44. In order to determine whether this novel ligand for NKp44 is activating or inhibitory, NK cell killing of astrocytes and IFN-γ production was tested with and without the blocking of NKp44 interactions. Blocking NKp44 interactions decreased NK cell killing of astrocytes and IFN-γ production, implicating NKp44 interaction with NKp44L on astrocytes activates NK cell function.

Both astrocytes and NK cells play important roles in HIV-1 infection. We studied the context of HIV-1 infection in the CNS using a highly conserved HIV gp41 3S peptide that is essential for HIV-1 entry into target cells. We evaluated NKp44L expression on astrocytes, NK cell killing of astrocytes, and NK cell production of IFN-γ in the presence and absence of HIV-3S peptide stimulated astrocytes. Previous studies reported HIV-3S peptide induced the expression of NKp44L on T cells and expression of NKp44L increased with increasing concentration of HIV-3S peptide [14]. We stimulated astrocytes with increasing concentration of 3S peptide, and strikingly, found that NKp44L expression on astrocytes decreased with increasing HIV-3S peptide concentrations. This decrease in NKp44L expression decreased NK mediated cytotoxicity towards astrocytes, suggesting that NKp44 has a protective effect on astrocytes during HIV-1 infection. More studies will need to be conducted in order to determine the function of HIV-3S peptide on astrocytes. These modifications suggest NK cells may play a possible role in the pathogenesis of HAND by killing astrocytes. NK cells could either promote or limit HAND by their intrinsic immune responses. Based on animal models of CNS diseases, NK cells can have a protective or deleterious effect on CNS. NK cells can control CNS inflammation and autoimmunity by killing proinflammatory or autoreactive microglia [28, 29]. Other studies have shown that NK cells may have a deleterious effect on CNS during bacterial infection by inhibiting the dendritic cell activation [30]. However, it is not known whether these responses are mediated by brain resident NK cells or NK cells entering the brain during injury to the CNS. Future studies providing insights into the CNS NK cells and the cells that interact are highly warranted. The identification of NKp44 ligand on astrocytes and regulation of its expression by HIV 3S peptide is a promising step towards this direction. Many unanswered questions still remain, and the future challenge is to delineate the precise influence of NK cells on CNS pathology in context of HAND.

## Conclusions

In conclusion, we identified a novel ligand for NKp44 on astrocytes. Expression of this novel ligand decreased with increasing HIV-3S peptide concentration and blocking this novel ligand decreased NK cell killing. NK cell killing of astrocytes was decreased when astrocytes were incubated with HIV-3S peptide. From these studies, it could be predicted that during the course of HIV-1 infection of the CNS, NKp44L is decreased and leads to a protection of astrocytes from NK cell killing during HIV-1 infection of the CNS and could play a role in HAND.
